# The Position and Orientation of the Pulse Generator Affects MRI RF Heating of Epicardial Leads in Children

**DOI:** 10.1109/EMBC48229.2022.9871968

**Published:** 2022-07

**Authors:** Bhumi Bhusal, Fuchang Jiang, Daniel Kim, KyungPyo Hong, Michael C Monge, Gregory Webster, Giorgio Bonmassar, Laleh Golestanirad

**Affiliations:** Department of Radiology, Northwestern University, Chicago, IL 60611 USA.; Department of Biomedical Engineering, Northwestern University, Evanston, IL 60608 USA.; Department of Radiology, Northwestern University, Chicago, IL 60611 USA.; Department of Radiology, Northwestern University, Chicago, IL 60611 USA.; Department of Pediatrics (Cardiology), Feinberg School of Medicine, Chicago, IL 60611 USA.; Department of Pediatrics (Cardiology), Feinberg School of Medicine, Chicago, IL 60611 USA.; A. A. Martinos Center for Biomedical Imaging, Massachusetts General Hospital, Boston, MA, USA; Department of Radiology and Department of Biomedical Engineering, Northwestern University, Chicago, IL 60611 USA.

## Abstract

Infants and children with congenital heart defects often receive a cardiac implantable electronic device (CIED). Because transvenous access to the heart is difficult in patients with small veins, the majority of young children receive epicardial CIEDs. Unfortunately, however, once an epicardial CIED is placed, patients are no longer eligible to receive magnetic resonance imaging (MRI) exams due to the unknown risk of MRI-induced radiofrequency (RF) heating of the device. Although many studies have assessed the role of device configuration in RF heating of endocardial CIEDs in adults, such case for epicardial devices in pediatric patients is relatively unexplored. In this study, we evaluated the variation in RF heating of an epicardial lead due to changes in the lateral position and orientation of the implantable pulse generator (IPG). We found that changing the orientation and position of the IPG resulted in a five-fold variation in the RF heating at the lead’s tip. Maximum heating was observed when the IPG was moved to a left lateral abdominal position of patient, and minimum heating was observed when the IPG was positioned directly under the heart.

## I. Introduction

Infants and children with congenital heart defects are often treated with cardiac implantable electronic devices (CIEDs) [1]. The device sends electrical signals to the heart muscle via elongated leads that are connected to an implantable pulse generator (IPG). Leads can either be routed to the heart’s interior intravenously, and then connected to an IPG that is placed in the pectoral region (endocardial system); or they can be directly stitched to the myocardium by opening the sternum and then connected to an IPG placed in the abdomen (epicardial system). Because endocardial lead implantation requires passing of the lead through subclavian vein which may not be feasible in infants and young children, both due to small vein size and congenital venous anomalies, children and adults with congenital heart disease receive epicardial CIEDs [2].

Unfortunately, once epicardial leads are implanted, the patient is no longer eligible to receive magnetic resonance imaging (MRI) exams. This is because electric and magnetic fields generated by MRI scanners can interact with conductive leads and amplify the specific absorption rate (SAR) of radiofrequency energy in the tissue around the tip [3-6]. This in turn can cause excessive tissue heating and potential thermal injuries. Although MR-conditional endocardial CIEDs have been approved by the FDA, no such labeling currently exists for epicardial CIEDs. Similarly, many studies have explored safety of MRI in patients with non-conditional endocardial CIEDs, but safety of MRI in children with epicardial leads is less explored [7-9].

It is established that the trajectory and configuration of an electronic implant strongly affects its MRI-induced RF heating [10-14]. From chest x-ray images of patients with CIEDs reported in the literature, one can observe that there are more variations in the configuration of epicardial CIEDs compared to endocardial devices. This is partially due to the fact that in endocardial CIED implantation, the IPG is consistently placed in the subpectoral pocket and leads are passed through the subclavian vein, which limits the variation in their trajectory. In contrast, there are few firm rules or guidelines for placing epicardial systems. As a result, surgeons position the IPG and leads based on prior experience and best practices handed from surgeon-to-surgeon, leading to substantial variation in lead trajectories and IPG orientations.

In this work, we examined the degree to which the position and orientation of the IPG affected RF heating of an epicardial CIED implanted in a pediatric anthropomorphic phantom. We configured the device following a common clinical practice, where the excess length of the lead was looped on the anterior surface of the heart to allow for growth, and the IPG was placed ~9 cm inferior to the center of the heart. We then systematically varied the lateral position of the IPG as well as its orientation while keeping the variation in the rest of the lead trajectory minimal. We assessed whether certain IPG positions were favorable in terms of MRI RF heating.

## II. Methods

### A. Pediatric Phantom Preparation

**Figure 1:**
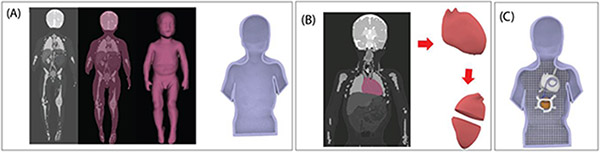
Steps in creating the pediatric phantom. (A) Segmented MR images were used to create the body silhouette, which was then morphed to create a 3D model of a torso-shaped container. (B) A heart-shaped mold was created such that it could be filled up with agar-based solution and opened once the gel solidified. (C) The assembled phantom.

A human shaped head and torso phantom representing a pediatric patient was designed based on segmented MR images of a 29-months-old child [15] (Fig. 1). MRI masks were imported into 3D-slicer (Slicer 4.10, http://slicer.org), where we used segment editor module to create 3D surface models of the body silhouette and the heart. Models were further processed using a CAD tool (Rhino 6.0, Robert McNeel & Associates, Seattle, WA) to create 3D printable objects representing a torso-shaped container and a heart-shaped mold which was designed to be filled up with agar-based gel mimicking heart tissue. Additionally, we designed and printed grids that fitted the inner surface of the phantom to help positioning the trajectory of CIED leads along desired paths. 3D models were printed with a stereolithography printer (Form 3L, Formlabs, Somerville, MA) using rigid resin. The experimental procedures involving data from human subjects were approved by the Institutional Review Board.

To fill the interior of the phantom, polyacrylic acid (PAA) gel was prepared by mixing Polyacrylic acid partial sodium salt (436364, Sigma-Aldrich Inc, St. Louis, MO, USA), with salt (NaCl) and distilled water, based on the ASTM standard [16]. The mixture was homogenized by using a blender. The gel had electrical conductivity of σ = 0.47 S/m and relative permittivity of ε_r_ = 88 representing properties of average tissue. A heart-shaped agar-based semi-solid gel was prepared by mixing agar powder (40 g/L) with saline (4 g/L) while slowly applying heat until the solution reached the boiling temperature. The gel was then poured into the heart mold and left to solidify as it cooled down to the room temperature. The final semi-solid gel had electrical conductivity of σ = 0.69 S/m and relative permittivity of ε_r_ = 86, representing properties of heart tissue.

### B. CIED Device Configurations

RF heating measurements were performed with a 35 cm epicardial lead (Medtronic CapSure^®^ EPI 4965, Medtronic Inc., Minneapolis, MN) connected to a Medtronic IPG (Azure^™^ XT DR MRI SureScan). A high resolution (0.01 °C) MR compatible fluoroptic temperature probe (OSENSA, Vancouver BC, Canada) was attached to the tip of the lead to measure temperature rise during MRI scan. The tip of the lead with the probe was inserted into the heart in a position resembling stimulation of left-ventricular region. The lead was then routed toward the IPG, making a loop on the anterior surface of heart. Remaining length of the lead was looped around the IPG.

**Figure 2:**
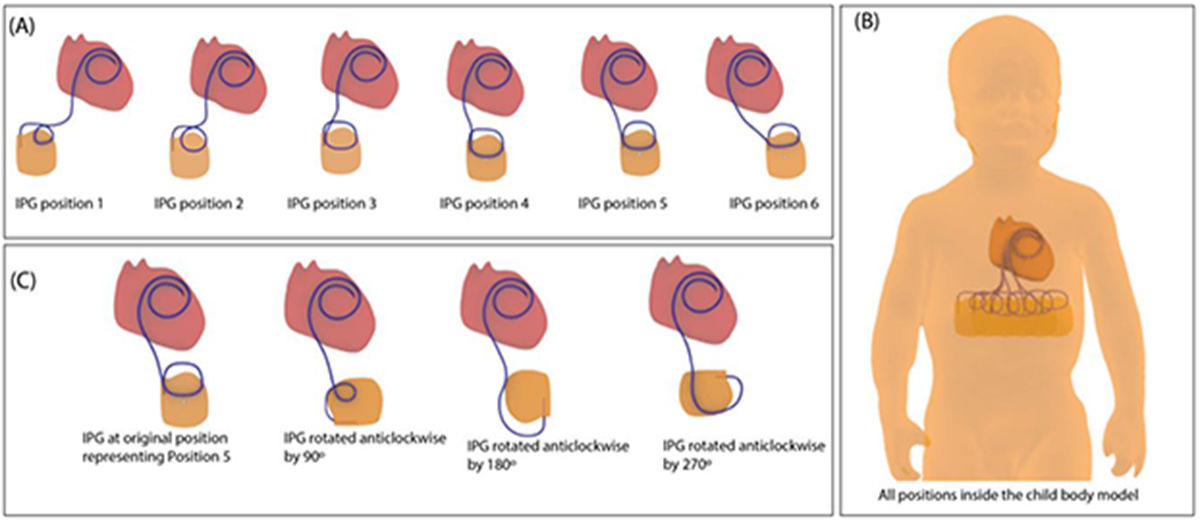
(A) 3D models of CIED lead and IPG configurations for six different positions of IPG moving from right to left of patient abdomen. (B) The CIED configurations shown together inside the body model from which the torso phantom was prepared. (C) CIED configurations with IPG at position 5 with four different orientation of the IPG.

**Figure 3:**
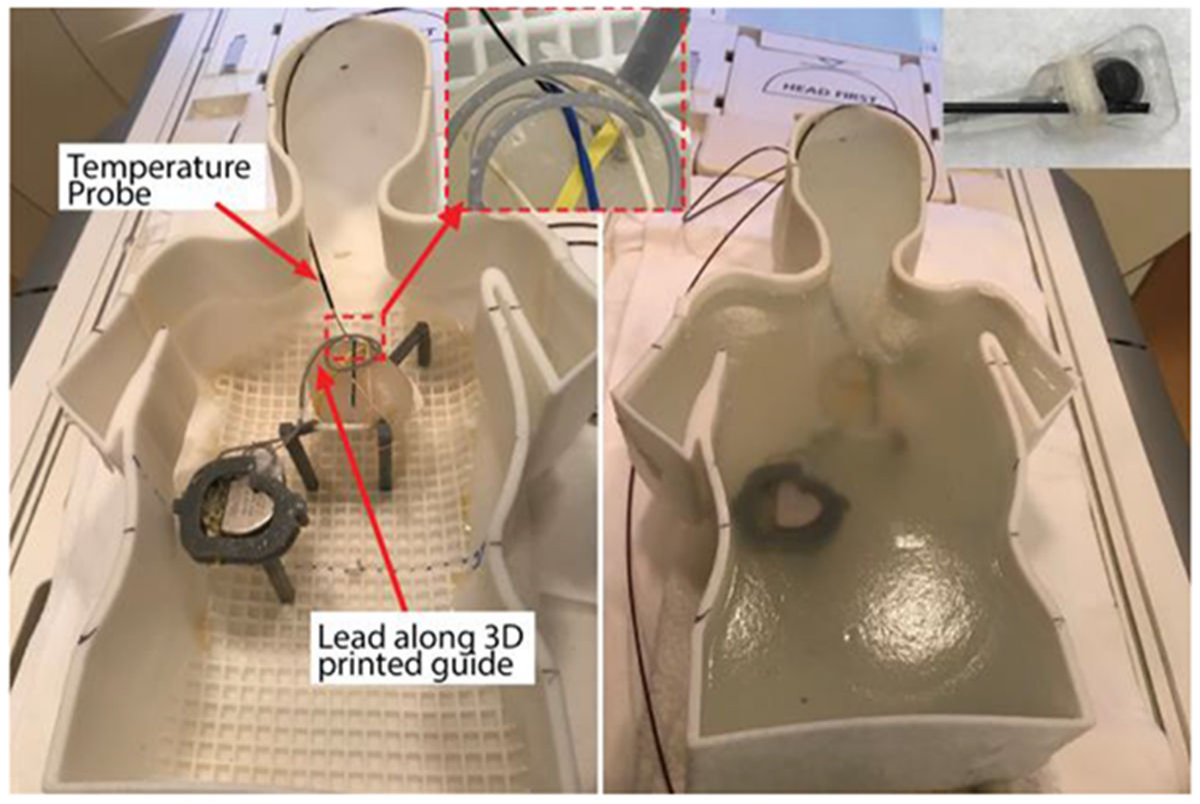
Experimental setup. CIED lead was configured with the help of a 3D printed trajectory guide, shown before and after filling the gel phantom. The insets show the close view of temperature probe connected to lead tip and lead-probe insertion into heart mimicking gel.

The IPG was first positioned in the abdomen at the vertical distance of ~ 9 cm inferior to the center of the heart. The lateral position of the IPG was then varied from right to left at ~2 cm increments for a total of 6 positions (Fig. 2). Additionally, at each position, we rotated the IPG (which remained in a coronal plane) in 90° increments (Fig. 2 & Fig. 3) as different IPG orientations are common in clinical practice.

### C. RF Exposure and Temperature Measurements

RF heating experiments were performed in a Siemens 1.5 T Aera scanner, using the body coil for both transmit and receive. The phantom was positioned inside the scanner such that the heart was at the isocenter of the scanner. A high SAR steady state free precision (SSFP) sequence (Repetition time (TR) = 39 S; Echo time (TE) = 1.69 ms; Field of view (FOV) = 300 mm; Acquisition time (TA) = 5:11 min; Flip Angle (FA) = 80°) was used for the RF heating measurements. This sequence is one of the commonly used sequences in cardiac imaging. The scanner reported root mean square value of B_1_ (B_1_^+^rms) was 4.7 μT. The setup for the RF heating experiment has been shown in Fig. 3. All the RF heating measurements were performed with the device in stimulation off mode.

## III. RESULTS

**Figure 4:**
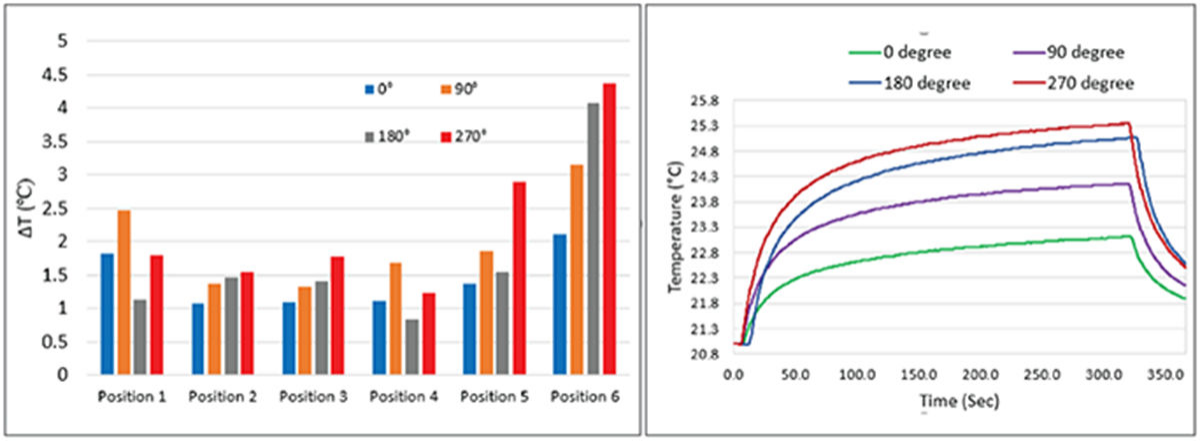
Temperature rise at the epicardial lead tip for different positions and orientations of IPG (Left). The temporal variation of temperature at the lead tip for four different orientations of IPG placed at position 6 also shown (Right).

The measured temperature rise is given in Fig. 4. The position and orientation of the IPG substantially affected RF heating of the lead at the tip. Temperature rise at the tip varied from 0.84°C to 4.36°C, representing a five-fold change in RF heating. Shifting the IPG’s lateral position while keeping its orientation constant resulted in approximately four-fold variation in the RF heating. Less variation was observed due to the change of IPG’s orientation alone. Overall, configurations with the IPG closer to the left wall of the phantom resulted in higher heating. This is consistent with earlier studies that reported higher RF heating for implants closer to the left side of phantom compared to right side [17, 18].

## IV. DISCUSSION AND CONCLUSIONS

When it comes to the RF heating of electronic implants during MRI, the configuration of the device within the patient’s body and the trajectory of the leads in particular, are shown to substantially affect the outcome [14, 19, 20]. This is because the magnitude of the tangential component of MRI incident electric field along the trajectory of an implanted lead is a strong determinant of its RF heating [21-23]. The changes in IPG’s position and orientation affects the trajectory of segments of the lead that are directly attached to it. In this study, we tried to minimize the variation of the lead trajectory around the heart while varying the IPG’s position and orientation. However, small segments of the lead trajectory at the interface with the IPG needed to be adjusted in accordance to the changes in IPG’s position/orientation. This caused those portions of lead to be exposed to a different electric field (as the electric field distribution is not homogenous inside the body) which in turn contributed to the variation in the RF heating. Additionally, the rotation of IPG leads to change in the loop configuration around IPG, which are supposed to affect the RF heating behavior as reported in earlier studies [3, 24]. The general trend in the result indicate that the configurations with IPG position close to left edge of phantom abdomen are prone to produce higher heating compared to those near the center or on the right side. This is consistent with the earlier studies, where the RF heating was reported to be higher at left side of phantom compared to right side [17, 18]. The higher heating at left side can be explained by considering electric field distribution inside the phantom. The electric field is generally smaller near the center of phantom and increases as one moves toward the edges. Moreover, due to the elliptical polarization of fields in human body, the upper surface of the phantom will have higher electric field on left side compared to right side, leading to higher heating in the implants on the left side [17, 18].

The findings of this study suggest that, the IPG position and orientation may be a factor to be considered while evaluating safety of using MRI in patients with epicardial leads. In the current exploration of lateral-medial IPG positioning, positions where the lateral borders of the IPG are within the lateral borders of the heart mass were associated with reduced RF heating risks. While additional work is required to understand the three-dimensional implications of IPG positioning, these preliminary results demonstrate the value of phantom modeling to improve surgical positioning for reducing RF heating risks. Furthermore, this study includes a limited number of device configurations and a single lead model. More work is required to draw more general conclusions related to IPG positioning.

## Clinical Relevance—

This study examines the role of device configuration on MRI-induced RF heating of an epicardial CIED in a pediatric phantom. Results could help pediatric cardiac surgeons to modify device implantation to reduce future risks of MRI in patients.
